# Silk fibroin/poly (vinyl alcohol) blend scaffolds for controlled delivery of curcumin

**DOI:** 10.1093/rb/rbv008

**Published:** 2015-05-26

**Authors:** Xiaomeng Li, Jinli Qin, Jun Ma

**Affiliations:** ^1^Advanced Biomaterials and Tissue Engineering Center, Huazhong University of Science and Technology, Wuhan 430074, China; ^2^Department of Biomedical Engineering, School of Life Science and Technology, Huazhong University of Science and Technology, Wuhan 430074, China

**Keywords:** silk fibroin, hydrogel, curcumin, controlled delivery

## Abstract

A silk fibroin/poly (vinyl alcohol) porous scaffold with a water vapor transmission rate of 2125 ± 464 g/m^2^/day has been developed via thermally induced phase separation (gelation) and freeze-drying process. A hierarchical architecture of micropores and nanofibers was observed inside the scaffolds, and the related structures were analyzed. The viability and proliferation of 3T3 fibroblasts were examined, which indicated that the scaffolds exerted low cytotoxicity. After loading curcumin, the scaffolds can suppress the growth of 3T3 fibroblasts. The release behavior of curcumin from the scaffolds was investigated. At pH = 7.2, the release profiles showed no significant difference for the loading amounts of 0.5 mg and 0.25 mg per sample. Meanwhile, the cumulative amount of released drug at pH = 5.7 was significantly more than that in neutral solution due to more degradation of the scaffolds. It was suggested that the silk fibroin/poly (vinyl alcohol) blend scaffolds could be potentially used as wound dressing materials.

## Introduction

Biodegradable polymers, including natural proteins, have been widely studied as biomaterials for the medical applications [1–4]. Silk fibroin (SF), which is a natural protein generated from silk cocoons [5], can be fabricated into various biomaterials for wound healing [6–8], tissue regeneration [9, 10] and drug delivery [11–13]. Porosity of biomaterials is one of the essential elements for tissue repair and regeneration [14]. These SF-based porous scaffolds could be fabricated by blending SF with collagen [15, 16], chitosan [17] and/or other biocompatible materials [18, 19]. Controlled delivery of drug or growth factors is important to the design of biomaterials. Usually, drugs can be loaded into porous and/or fibrous biomaterials by adsorption. Because many drugs have good affinity to SF, SF-based drug delivery systems have been studied extensively. For example, the porous SF hydrogels made by lyophilization could provide a sustained delivery system of antibodies for over 38 days [11]. The photo-crosslinked SF/PVA hydrogels were investigated as a delivery system of macromolecular drugs [13]. It is known that curcumin is an anti-cancer, anti-oxidant and anti-inflammatory drug [20]. The curcumin-loaded nanosized hydrogels were able to deliver drugs into cancer cells [21–24]. The curcumin-loaded SF scaffolds were fabricated via a freeze-thaw method, and the obtained porous scaffolds demonstrated a slow and sustained release profile of curcumin [25]. Furthermore, curcumin could be linked to the backbone of the hydrogel and the resultant hydrogels could be used as soft tissue fillers after tumor removal [26].

Nanofibers could provide better environments for cell growth and tissue regeneration [16, 27, 28]. An attractive method for fabrication of nanofibrous scaffolds is thermally induced phase separation [29], which mimics the natural extracellular matrix well. Poly (vinyl alcohol) (PVA) could offer attachment of signal molecules or drugs via hydroxyl groups on the backbone. PVA blended with SF could be fabricated into novel biomaterials, such as SF-based particles with various appearances [30, 31].

The aim of this study is to develop a novel porous SF/PVA scaffold with nanofibrous structures by thermally induced phase separation. The gelation process of SF induced by phase separation could lead to the formation of stable hydrogels with high mechanical strength and toughness [13]. In this study, the morphology, structure, water uptake, water loss, vapor transmission properties and cytotoxicity of the obtained SF/PVA blend scaffolds were investigated. The release behavior of curcumin from the blend scaffolds was also studied.

## Materials and Methods

### Materials

PVA (Type 1788, 87.0∼89.0% hydrolyzed) was purchased from Aladdin Reagent Company (China) and curcumin was from Alfa Aesar (Tianjin) Chemical Co. Ltd. (China). Silk cocoons were bought from Huazhong Agricultural University (Wuhan, China). The viscose spunlace non-woven fabrics were purchased from Fenglin Non-Wovens Co. Ltd. (ZhengZhou, China). The thickness of the fabrics was 0.4 mm and the surface density was 62 g/m^2^. All other chemical agents were of chemical grade or above and used as received.

### Preparation of the SF/PVA blend scaffolds

SF was obtained by removing silk sericin from silk cocoons [17]. In brief, silk cocoons were treated in boiled 0.5% Na_2_CO_3_ solution for 30 min and washed using distilled water. The obtained products were dried in oven at 37°C. The CaCl_2_/C_2_H_6_O/water system (molar ratio = 1:2.5:8) was used to dissolve SF (0.03–0.05 g/ml) and the purification was carried out via dialysis against distilled water for 3 days (molecule weight cutoff, MWCO = 3500). SF was lyophilized at −55°C for 24 h, and the drying was performed at room temperature and 5–20 Pa. The SF powder cakes were collected and stored at 4°C for the following experiments.

The PVA powder was added to distilled water and then stirred at 80°C for full dissolution. After cooling down to room temperature, the SF powder was added to the PVA solution. The total mass in the solution was about 10%. After stirring overnight, the mixtures were poured into the molds (six-well culture plates). Subsequently, a piece of non-woven cellulose fabrics with the same diameter of the mold well was placed in the middle of the solution. After setting for 24 h at room temperature, the SF/PVA mixtures changed to hydrogels. The SF/PVA hydrogels with different SF/PVA weight ratios (5:5, 4:6, 3:7 and 2:8) were frozen at −20°C and subsequently lyophilized at −55°C for 24 h. The drying was performed at room temperature, 5–20 Pa. The obtained blend scaffolds were stored in plastic bags at 4°C.

### Characterization of SF/PVA blend scaffolds

#### Mechanical performance

The mechanical properties of the freeze-dried blend scaffolds (1.5 cm in diameter) were investigated using a universal mechanical analyzer (AG-100KN, Shimazu, Japan). For compression testing, the samples were compressed to about 70% of their original height with a constant crosshead speed of 0.005 mm/s at room temperature.

#### Morphology and structure

The dried samples were cut into small pieces, mounted onto the specimen holder and were subsequently sputter coated using gold for better conductivity. The cross-section and surface were observed by scanning electron microscopy (SEM, NOVA Nano SEM 450, FEI, USA). For the structural analysis, the samples were measured using Fourier transform infrared spectroscopy (FT-IR, Nexus Thermo Nicolet, USA) in the reflected mode, in the wavenumber range of 4000–550 cm^−^^1^. Fourier self-deconvolution of the spectra covering the amide I region (1590–1710 cm^−^^1^) was carried out as previously described [11]. The relative areas of each single band were used to calculate the fraction of the secondary structures.

#### Swelling behaviors and water uptake capability

The dried SF/PVA scaffolds were immersed in water. The scaffolds were taken out at certain time intervals, and their weights were recorded after removing the excess water by filter paper. The degree of water uptake was plotted versus the time.

Fluid content (%) = (*W*_w_ − *W*_0_)/*W*_w_ × 100%, where *W*_w_ is the wet weight, and *W*_0_ is the initial dry weight. The measurements were done in triplicate.

#### Water evaporation rate

The above swollen scaffolds were kept in an incubator at 37°C and 35% relative humidity. The weight loss versus time was recorded at regular time intervals.

Weight remaining (%) = *W_t_*/*W*_w_ × 100%, where *W*_w_ is the weight at the initial time and *W_t_* is the weight at time point of t.

#### Water vapor transmission rate

The water vapor transmission rate (WVTR) was measured to evaluate the moisture permeability of the blend scaffolds. Each sample was cut into a round shape and mounted onto a bottle which contained 10 g of water at the initial time. All the samples were placed in an incubator at 37°C and 35% relative humidity. The weights of the samples were recorded at certain time intervals and the weight loss versus time was linearly fitted to calculate the WVTR value.

WVTR = S × 24/A (g/m^2^/day), where S is the slope (g/h) and A is the tested area in m^2^.

#### Release of curcumin from curcumin-loaded SF/PVA scaffolds

In each SF/PVA scaffold, 0.5 mg of curcumin was loaded. During the drug loading process, the curcumin powder was firstly dissolved in 0.1 ml ethanol and added into 1 ml phosphate buffer saline (PBS) solution (pH = 7.2) with 0.5% Tween 80. Subsequently, the scaffolds absorbed the curcumin solution in the incubator at 37°C. For the study of different drug loading amounts, 0.25 mg of curcumin-loaded samples were also prepared.

Because of the limited solubility of curcumin in water, the release medium for curcumin was 0.2 M PBS with 10% ethanol and 0.5% Tween 80. The release solutions with different pH values were prepared by varying the ratio of 0.2 M sodium di-hydrogen phosphate solution (solution A) and 0.2 M sodium hydrogen phosphate (solution B). For the PBS buffer at pH 7.2, 28 ml solution A and 72 ml solution B were mixed, and the final solution was adjusted using a pH meter. For pH = 5.7, 93.5 ml solution A and 6.5 ml solution B were mixed. All the buffer solutions were prepared, and the pH value was validated at room temperature.

For the release studies, each curcumin-loaded SF/PVA scaffold was immersed in a tube containing 40 ml release medium. The samples were stirred at 100 rpm and 37°C. At certain time intervals, 0.2 ml of the sample solution was taken out and an equal amount of fresh release medium was refilled. The concentration of curcumin in the sample solution was measured using an automatic microplate spectrophotometer (Eon, Biotek, USA) at the wavelength of 426 nm. The absorbance values were converted to drug release amounts according to the calibrated curve. The drug release amounts were normalized to the drugs initially loaded on the SF/PVA scaffolds. For all samples, the drug release profiles were measured until the equilibrium reached. The SF/PVA blend scaffolds without loading curcumin were used as a control group. All the experiments were performed in triplicate. One-way analysis of variance was performed to compare the data and *P* < 0.05 was considered to be statistical significance. After immersed in the release medium for 5 days, the SF/PVA scaffolds were taken out and freeze-dried. The dried samples after the release assays were observed by SEM.

#### Cytotoxicity evaluation.

The NIH 3T3 mouse embryonic fibroblast cells were cultured in Dulbecco’s Modified Eagle’s Medium (DMEM, Giboco), 10% fetal bovine serum (FBS, HyClone), 100 U/ml penicillin and 100 μg/ml streptomycin at 37°C in a humidified atmosphere of 5% CO_2_. The cytotoxicity tests of the blend scaffolds were performed by the direct contact method. Before culturing, the SF/PVA scaffolds were sterilized under an ultraviolet light overnight. The scaffolds were placed on the bottom of 48-well plates for cell seeding. The blank wells were used as control. In each well, 400 μl cell suspension (4 × 10^4^ cells/ml) was used. The culture medium was changed everyday. After incubating the cells for 1, 3, 5 and 7 days, the plates were taken out for cell viability evaluation using Cell Counting Kit 8 (CCK8) assay. In each well, 40 μl of CCK8 solution was added. After the incubation at 37°C for 2 h, 100 μl of the supernatant was taken out and placed in 96-well plate for optical density measurements using a microplate spectrophotometer (Eon, BioTek instruments, USA). Because curcumin can interfere the measurement using CCK8 assay, an extraction method was adopted to study the cytotoxicity of the blend scaffolds with and without curcumin. Briefly, 0.5 g of sample was placed in 20 ml of culture medium at 37°C for 24 h in an incubator. After the cells (2 × 10^4^ cells/ml) were seeded and incubated for 24 h, the medium in each well was replaced by the extraction medium. The cells cultured using the normal culture medium acted as the control. After another 1, 2 and 3 days of incubation in the extraction medium, the cell viability was measured using CCK8 assay.

## Results and Discussion

### Preparation of SF/PVA blend scaffolds

**Figure 1.**
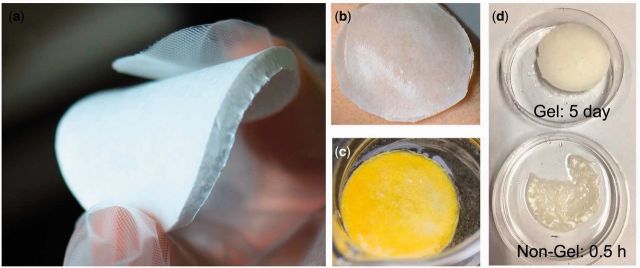
(**a**) Photos of the SF/PVA blend scaffolds, (**b**) the swollen hydrogel scaffold on the skin, (**c**) the sample after immersed in the release medium for 5 days and (**d**) the effect of gelation process

The dried SF/PVA blend scaffolds had white color and showed a uniform surface. The thickness of the scaffolds was about 3 mm. In the dried scaffolds, the weight ratio of the fabrics was about 17%. The samples were very flexible and they could be bended by hand (Fig. 1a). The wet scaffold was easy to adhere onto the skin surface and the shape of the wet one was able to fit the curved skin surface (Fig. 1b). In addition, after immersed in PBS buffer solution for 5 days, the sample still maintained the shape (Fig. 1c). The yellow color of the sample was from curcumin loaded in the blend scaffold. Without chemical cross-linking, the gelation process endowed stability to the porous scaffolds. In comparison with the blend scaffold with gelation process, the sample without gelation dissolved after 0.5 h of immersion in water as shown in Fig. 1d.

**Figure 2.**
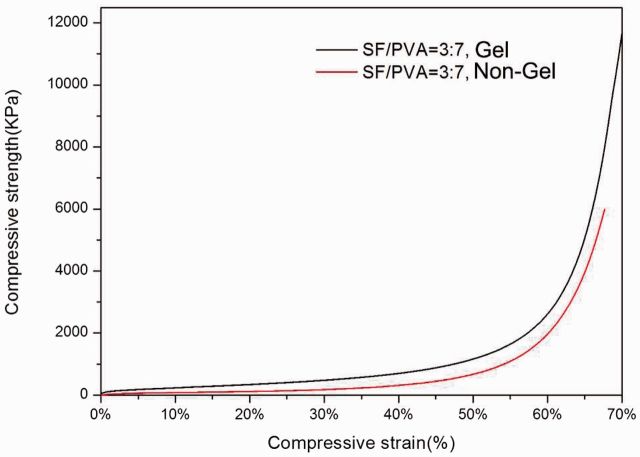
Compressive stress–strain curves for SF/PVA blend scaffolds with and without gelation process

The compressive stress–strain curves for the SF/PVA (7:3) blend scaffolds with and without gelation process are shown in Fig. 2. Mechanical testing of the scaffolds under compression indicated that the scaffold after gelation process was stronger and more robust than that without gelation. For compressive strain of 20%, 30% and 50%, the compressive strength increased by factors of 2.85, 2.49 and 1.29 after gelation process. The improvement in mechanical properties is consistent with the above observations.

### Morphology of SF/PVA blend scaffolds

**Figure 3.**
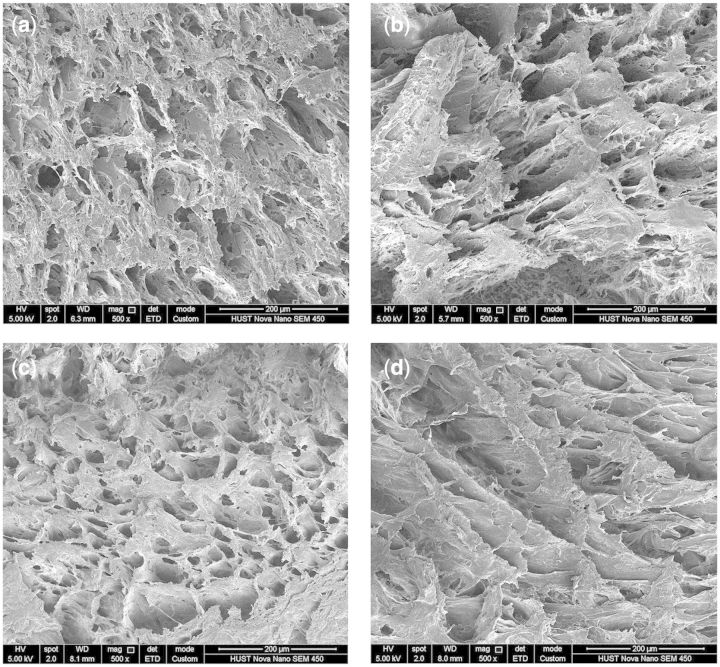
SEM images of the cross sections of SF/PVA blend scaffolds with different weight ratio (**a**) 5:5, (**b**) 4:6, (**c**) 3:7 and (**d**) 2:8

The morphology of the cross section and outer surface of the SF/PVA blend scaffolds was investigated by SEM. The SEM results demonstrated that all the samples with different ratios of SF and PVA had three-dimensional porous structures. According to the SEM images of the cross sections (Fig. 3), the porous structures changed depending on the SF/PVA ratios. The pore size was found to be in a wide range from several micrometers to more than 100 μm. From the SEM images, the pore size distribution of the samples with SF/PVA ratio of 3:7 seemed more uniform than that of the others.

**Figure 4.**
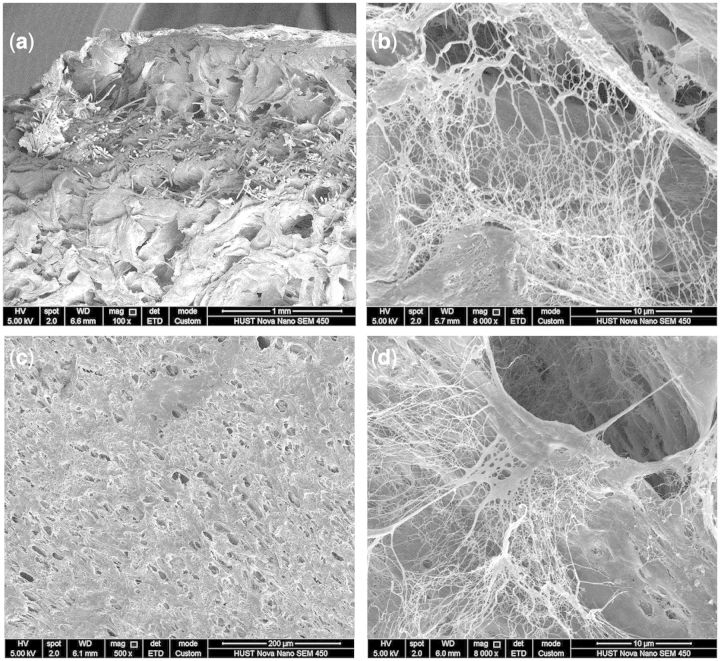
SEM images of SF/PVA blend scaffolds with SF/PVA = 3:7 in the cross section (**a**, **b**) and surface (**c**, **d**)

As shown in Fig. 4a, the cellulose fibers were surrounded by the SF/PVA blends. The magnification on the cross-section showed nanofibrous structures, especially inside the pores (Fig. 4b). As shown in Fig. 4c, the surface of the SF/PVA blend scaffolds exhibited a homogenous, dense porous structure. The pore size on the surface seemed much smaller than that of the interior section (Fig. 4c). This characteristic would make the scaffolds more suitable for wound healing [32]. Nanofibrous structures were also found inside the pores on the surface (Fig. 4d). The existence of the small pores and nanofibers could limit the diffusion of drug molecules and regulate the release behavior of the drugs.

### Structural characterization

**Figure 5.**
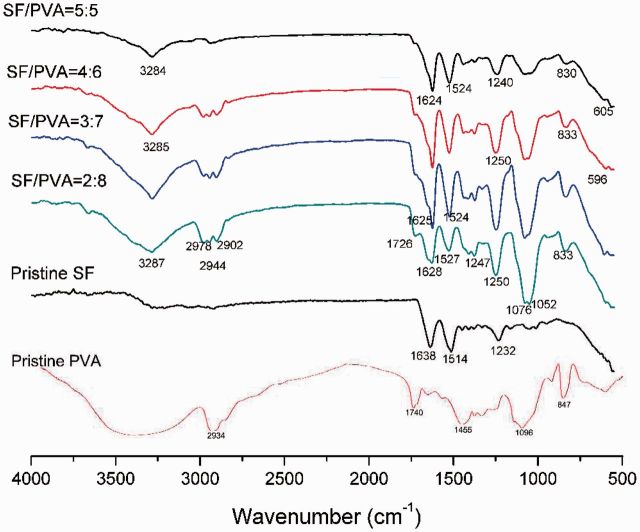
FT-IR spectra of the SF/PVA blend scaffolds and pristine SF and PVA powders

The FT-IR spectra of the SF/PVA scaffolds (top surface) with different ratios were examined to measure the structural changes after the process of blending and gelation, as shown in Fig. 5. All the SF/PVA scaffolds have similar peaks. The bands at 3284–3287 cm^−^^1^ were attributed to the hydroxyl groups in SF and PVA. The bands at 2978 cm^−^^1^ were attributed to the saturated C-H groups (-CH_3_). The bands at 2944 cm^−^^1^ and 2902 cm^−^^1^ were attributed to C-H stretching vibrations (-CH_2_). The bands at 1726 cm^−^^1^ were assigned to the C=O groups. The bands at 1076 cm^−^^1^ and 1052 cm^−^^1^ were attributed by the C-O stretching mode. These bands assigned to PVA became stronger with increasing PVA contents. The bands at 830–833 cm^−^^1^ were attributed by the C-H bending mode. The small bands at 596–605 cm^−^^1^ were suggested to be attributed by the NH_2_ groups. The bands of amide I (1624–1638 cm^−^^1^) and amide II (1514–1527 cm^−^^1^) and amide III (1232–1250 cm^−^^1^) belonged to SF in the scaffolds. As can be observed, the position of the amide I, II and III bands shifted after blending with PVA, which implied that SF molecules had interactions with PVA.

For better understanding of the secondary structure changes, the contents of random coils, α-helices, β-sheets and turns were determined by the bands in the amide I region (1595–1705 cm^−^^1^) for both sides of the blend SF/PVA scaffolds [11, 33]. The frequency ranges of 1616–1637 cm^−^^1^ and 1695–1705 cm^−^^1^ were assigned to the enriched β-sheet structure in silk II form. The bands in the frequency range of 1638–1655 cm^−^^1^ were attributed to rand coils, 1656–1663 cm^−^^1^ to α-helices and 1663–1695 cm^−^^1^ to turns. According to the deconvolution results, 59.6 and 62.4% β-sheet structures were found on both side surfaces of the sample with SF/PVA = 3:7. For the samples with SF/PVA = 5:5 and 4:6, about 58.8% and 53.4% β-sheet structures were found on the top surface, whereas only random coils were found on the bottom surface. For the sample with SF/PVA = 2:8, 45.6% β-sheet structures were found on the top surface and 66.2% on the bottom surface. These results indicated that the structural uniformity of SF/PVA = 3:7 was the best one, which was in accordance with the SEM results, and thus the following experiments were carried out using the samples with SF/PVA = 3:7.

### Swelling properties and WVTRs

**Figure 6.**
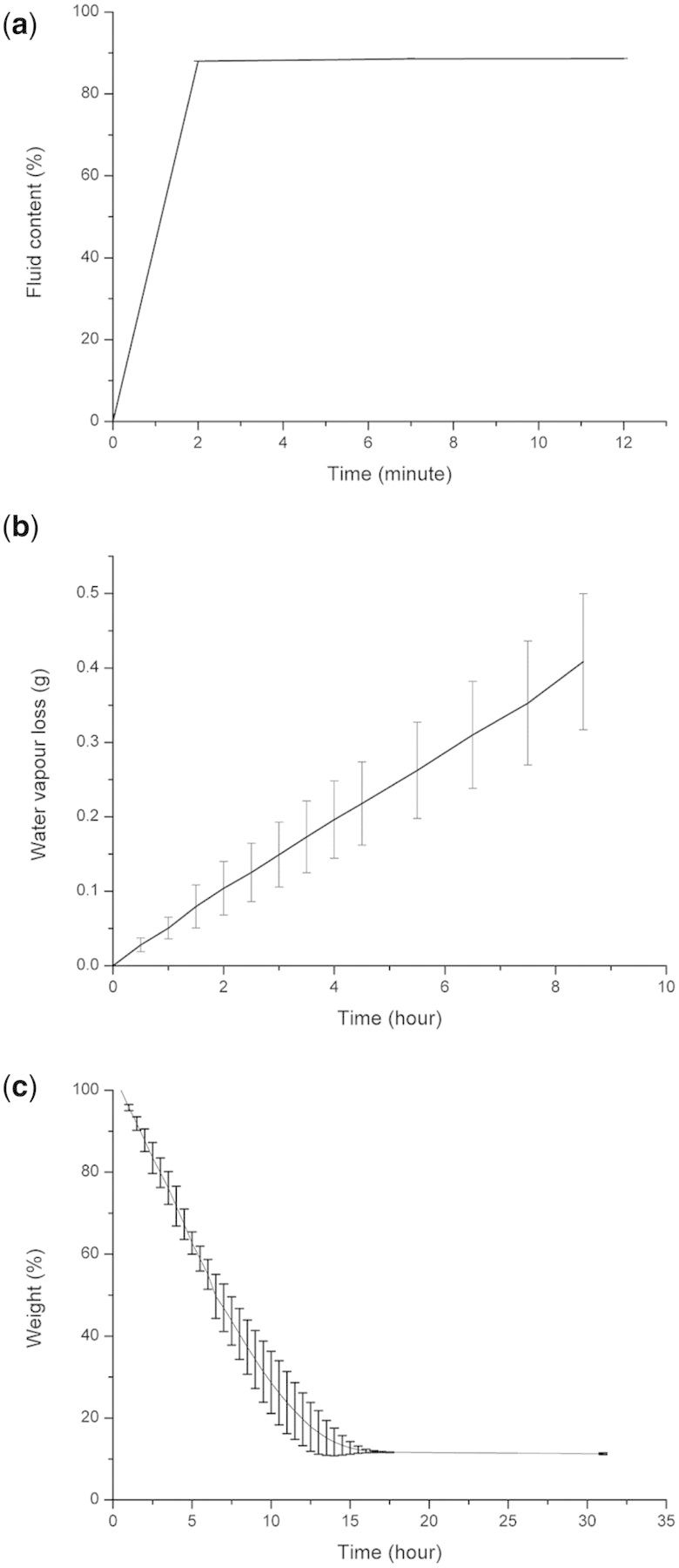
Water uptake measurements (**a**), water vapor transmitted across the samples (**b**) and water loss during dehydration (**c**)

The SF/PVA blend scaffolds (SF/PVA = 3:7) with a thickness of about 2.6 mm were investigated in the following tests. The degree of water uptake was measured at regular time intervals after immersion in water. As shown in Fig. 6a, the samples reached the equilibrium of fluid uptake in less than 2 min. The water content in the final swollen samples was about 88%. It was suggested that the SF/PVA scaffolds were able to uptake about 7.3 times weight of water. In addition, the swollen SF/PVA hydrogel scaffolds had a similar size to the dried one. According to the photo in Fig. 1c, the hydrogels did not change obviously in size and shape even after 5 days of incubation in PBS buffer.

For the measurements of WVTR, water vapor went through the porous SF/PVA structures in a linear rate. The calculated value of WVTR was 2125 ± 464 g/m^2^/day. In the previous study, the alginate/gelatin hydrogel dressing had a WVTR value of 2686 ± 124 g/m^2^/day [32]. The WVTR of another dextran hydrogel dressing was about 2280 g/m^2^/day [34]. It was suggested that an ideal rate of 2000–2500 g/m^2^/day of the wound dressings could provide a moisture environment for better wound healing effects [35]. The water loss was measured to be about 7%/h in the initial 10 h. Almost all the water evaporated from the swollen hydrogel scaffolds after 15 h. Herein, the developed SF/PVA blend scaffolds could meet the requirements of moisture wound healing principles and provide wound areas with suitable moist environment and evaporate wound leaks in time.

### Cytotoxicity of the SF/PVA blend scaffolds

**Figure 7.**
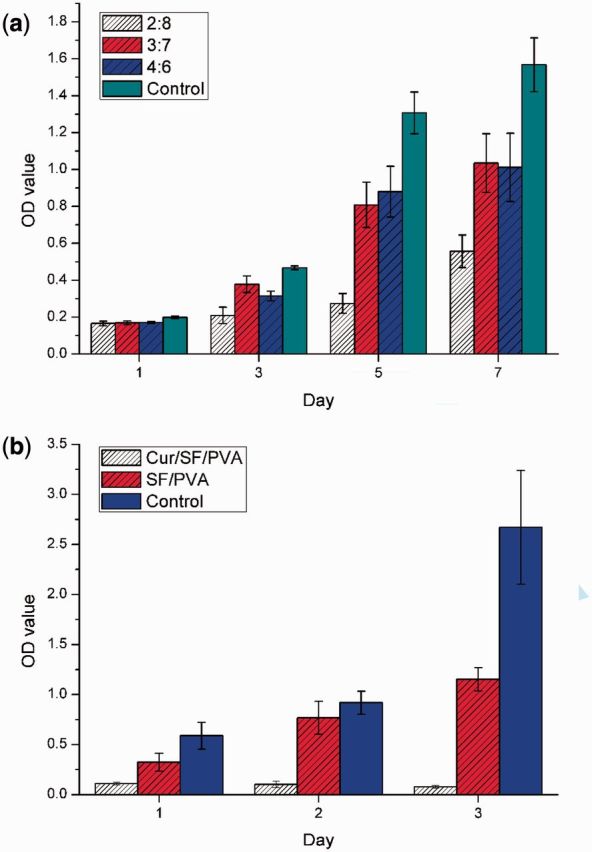
Cell culturing results of 3T3 fibroblasts (**a**) directly on the SF/PVA blend scaffolds and (**b**) extraction medium

The fibroblast cells were directly cultured on the SF/PVA blend scaffolds. From Fig. 7, the cells grown on the scaffolds continued to proliferate during the 7 days of cell culturing. The cell proliferation rate on the samples is lower than that on the blank plates because PVA does not support cell adhesion. There was no difference in the cell viability among the three samples on the first day. On day 3 after culturing, the cells on the sample of 2:8 showed the lowest cell density, whereas the cell number on the sample of 3:7 was the highest. On days 5 and 7, the cell viability on the samples of 3:7 and 4:6 was similar. Meanwhile, the cell density on the sample of 2:8 was much lower than the other two samples.

The extraction method was carried out to compare the cytotoxicity of the scaffolds with and without curcumin. The scaffolds without curcumin exerted low cytotoxicity, which is consistent with the results using the direct contact method. For the curcumin-loaded blend scaffolds, the viability of 3T3 fibroblasts decreased dramatically to about 19%, 11% and 2.9% of the blank control on days 1, 2 and 3, respectively. Curcumin has selective toxicity toward cancer cells and not toward normal cells, because it can suppress the growth of tumor cells by suppressing NF-κ B regulated gene [24]. It is suggested that the curcumin-loaded scaffolds can be used as soft tissue implants or dressings, especially after surgical treatments of tumors [25].

### Curcumin release from the SF/PVA blend scaffolds

The standard curves of the release medium containing curcumin were linear from 0.125 to 1.25 μg /ml. All the cumulative release amounts were normalized to the drug loading amounts. According to the results of the control groups without curcumin loading, the absorption values at 426 nm had no difference compared with that of blank medium without curcumin. It was indicated that the scaffold degradation had no influence on the curcumin measurements.

**Figure 8.**
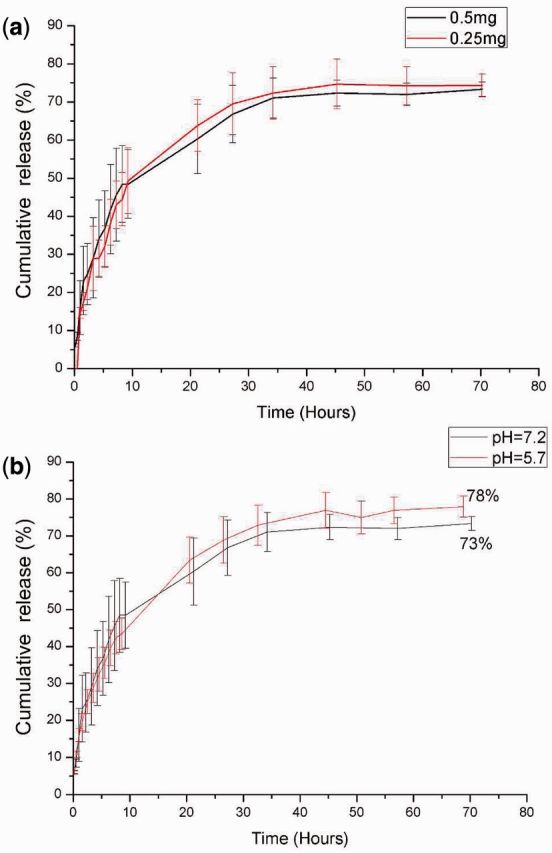
Curcumin release profiles in ethanol-PBS-Tween80 release medium with different drug loading amounts at pH = 7.2 (**a**) and different pH values (**b**)

As shown in Fig. 8, the release behavior of the drugs from the blend scaffolds demonstrated a two-stage profile. For different drug loading amounts, no statistically significant difference (*P* > 0.05) was found during the release assays, as shown in Fig. 8a. The reduction of drug loading amount to 0.25 mg per sample did not change the release rate.

From Fig. 8b, it was observed that approximate 50% drugs came out from the scaffolds, and no significant differences were observed in the first 10 h (*P* > 0.05). After 70 h, 73% curcumin was released from the blend scaffolds at pH = 7.2. In the acidic environment (pH = 5.7), 78% curcumin was released from the blend scaffolds. For different pH values, the cumulative drug release amounts were found to be statistically different (*P* < 0.05). It was reported that the wound areas had an acidic pH value of about 5.7 in stage I wound, and the pH value would increase in stages II and III [36]. In this study, more drugs could be released from the resultant SF/PVA blend scaffolds in acidic environments, which could contribute to wound healing in stage I.

**Figure 9.**
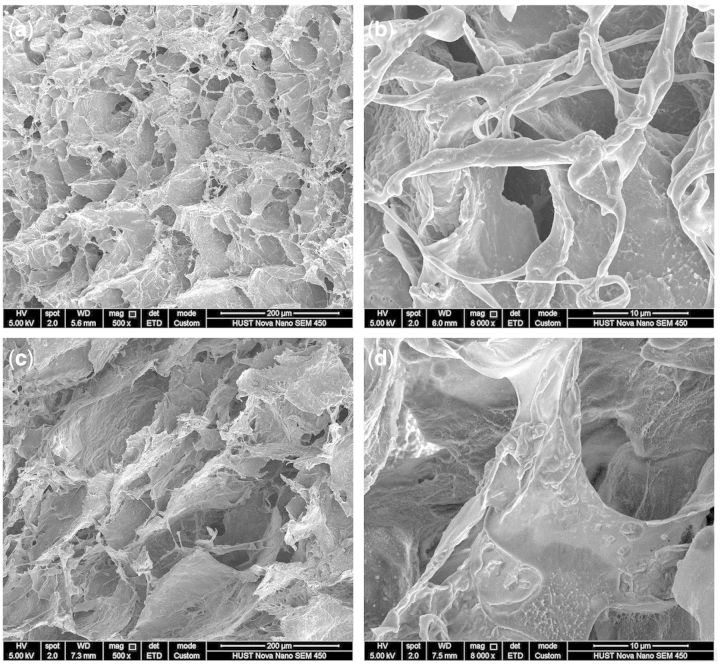
SEM images of the SF/PVA blend scaffolds after immersed in release medium for 5 days of the top surface (**a**, **b**) and the cross section (**c**, **d**)

The remaining drugs in the SF/PVA scaffolds were suggested to be released when the blend scaffolds degraded [12, 13]. The weights of the scaffolds were measured after the release experiments. At pH = 5.7, the weight loss of the freeze-dried scaffolds was 25.0 ± 0.9%, which was higher than that at pH = 7.2 (19.8 ± 2.2%). The difference in weight loss was statistically significant (*P* < 0.05). It was suggested that more curcumin could be released from the scaffolds because more degradation of the scaffolds occurred in acidic solution. After 5 days of immersion in release medium, the curcumin-loaded SF/PVA hydrogels were freeze-dried and the products were observed using SEM. From the SEM images in Fig. 9, the pores on cross-section and surface had no obvious changes. The nanofibers inside the pores disappeared due to the degradation of the blend scaffold. Meanwhile, fibrous patterns were observed on the walls of the pores. It was indicated that the degradation of SF/PVA occurred and the nanofibrous structures in the blend scaffolds began to be exposed. These nanofibrous structures could play the roles of extracellular matrix during tissue regeneration [11, 37]. Because no additional cross-linking agents were used in the fabrication, the obtained SF/PVA blend scaffolds and their degradation products were considered to be biocompatible [11, 27, 37]. Before the clinical applications, cell and animal experiments would be required to verify the safety and functions of the SF/PVA blend scaffolds.

### Mechanisms

**Figure 10.**
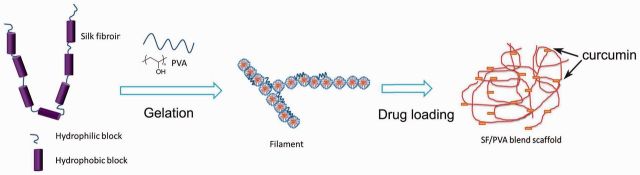
interaction mechanisms of self-assembly of SF nanofibers with PVA

The nanofibers found in the SF/PVA blend scaffold were considered to be related to the self-assembly of the SF molecules as shown in Fig. 10. There were large hydrophobic blocks and small hydrophilic blocks on the SF molecules. The SF molecules formed micelles by hydrophobic and hydrophilic interactions. The nanofibers were assembled by the small SF micelles. The hydrogen bond interactions between PVA and the hydrophilic blocks of SF could increase the rate of chain folding and help the transition of SF from random to crystal structures [38, 39].

Curcumin is hydrophobic and it mainly interacts with the hydrophobic domains of SF in the blend scaffolds. The slow and sustained release of curcumin from the blend scaffolds was determined by the non-covalent interaction between curcumin and SF [25]. The binding constant between SF and curcumin determined by fluorescence intensity changes was estimated to be 4.8 × 10^5 ^M^−^^1^ [25], which is much larger than that of curcumin to PVA (1.9 × 10^5 ^M^−^^1^) [40]. It was suggested that SF could bind more curcumin drugs than PVA. It could be possible to tune the release behaviors by varying the SF/PVA ratio in the blend scaffolds [13].

## Conclusion

In this study, we have described an approach for the creation of SF/PVA blend scaffolds with a hierarchical architecture of micropores and nanofibers via thermally induced phase separation and freeze-drying process. The obtained blend scaffolds exerted low cytotoxicity toward fibroblast cells. The curcumin release kinetics showed no relation to the loading amount. The cumulative release amount of curcumin was higher at pH 5.7 than that at pH 7.2, because the degradation of scaffolds was faster under acidic condition. The results suggested that the curcumin release rate could be controlled and the SF/PVA blend scaffolds could be potentially used as wound dressings.
